# Diagnosing malabsorption with systemic lipid profiling: pharmacokinetics of pentadecanoic acid and triheptadecanoic acid following oral administration in healthy subjects and subjects with cystic fibrosis 

**DOI:** 10.5414/CP201793

**Published:** 2013-01-29

**Authors:** Virginia A. Stallings, John T. Mondick, Joan I. Schall, Jeffrey S. Barrett, Martha Wilson, Maria R. Mascarenhas

**Affiliations:** 1 Division of Gastroenterology, Hepatology and Nutrition, The Children’s Hospital of Philadelphia,; 2 Department of Pediatrics, University of Pennsylvania Perelman School of Medicine,; 3 Division of Clinical Pharmacology and Therapeutics, The Children’s Hospital of Philadelphia, Philadelphia, PA, USA; 4 Pathology – Lipid Science, Wake Forest School of Medicine, Winston-Salem, NC, USA; * current address, Metrum Research Group, Tariffville, CT, USA

**Keywords:** dietary fat malabsorption, orlistat, cystic fibrosis, chronic pancreatitis, pentadecanoic acid, triheptadecanoic acid

## Abstract

Objective: A Malabsorption Blood Test (MBT) is proposed as an alternative method to the 72-hour stool and dietary collection for assessing the degree of fat malabsorption in people with pancreatic insufficiency. The MBT consists of a simultaneous oral dose of pentadecanoic acid (PA), a free fatty acid, and triheptadecanoic acid (THA), a triglyceride with three heptadecanoic (HA) saturated fatty acids requiring hydrolysis by pancreatic lipase before HA can be intestinally absorbed. The aim of this study is to demonstrate the ability of MBT to detect fat malabsorption in healthy adult subjects using the pancreatic lipase (PL) inhibitor Orlistat (Xenical^®^), and in subjects with CF and PI while on and off routine pancreatic enzyme doses. Materials and methods: The MBT with the PA and THA were delivered in a breakfast test meal (2.5 g PA and either 5 g or 8 g THA) to healthy adult subjects (ages 18 – 50 years, BMI 21 – 30) and to subjects with CF (> 12 years, FEV_1%_ predicted > 40%), after a 12-hour fast and 24 hours without dairy foods. Serum levels of PA and HA were assessed by gas-liquid chromatography, from blood samples drawn prior to MBT and then hourly for 8 hours. For healthy subjects, the MBT was administered before and after Orlistat treatment, and in subjects with CF, both with subjects receiving routine pancreatic lipase treatment (“on enzyme”) and also “off enzyme” treatment. Treatment groups were compared for baseline (C_0_) and maximum (C_max_) plasma concentrations of PA and HA over 8 hours: area under the curve (AUC) was calculated using linear trapezoid method. The ratio of HA to PA C_max_ and AUC was also calculated and compared. Results: For the healthy subjects (n = 15, 60% female, ages 21 – 49 years), absorption of HA was reduced 71% for C_max_ (p < 0.001) and 65% for AUC (p = 0.001) after Orlistat treatment, and absorption of PA was unchanged. For subjects with CF (n = 6, 50% female, ages 13 – 19 years), absorption of HA was minimal with subjects “off enzymes” and increased significantly with subjects “on enzymes” while absorption of PA did not differ between groups. Enzyme administration resulted in increased C_max_ HA/PA ratios from 0.02 to 0.92 and from 0.05 to 0.73 in subjects with CF receiving 5.0 g and 8.0 g of THA, respectively. AUC HA/PA ratios showed similar increases. Conclusions: In this pilot and feasibility proof-of-concept study, the MBT, utilizing the relative absorption of HA to PA, two odd-chained fatty acids, responds to changes in fat absorption in healthy subjects using a lipase inhibitor and in subjects with CF while on or off enzyme therapy. The MBT holds promise to provide a more accurate, specific and acceptable alternative to the 72-hour stool collection to quantify pancreatic-based fat malabsorption in a variety of clinical and research contexts.

## Introduction 

Optimum utilization of dietary fat requires a complex digestive process in which dietary fat moieties are hydrolyzed and then repackaged within the gut lumen for absorption and transport into the body. While lipolysis starts with gastric lipase, the primary enzyme of fat digestion is pancreatic lipase. Dietary fats are digested into free fatty acids and monoglycerides which then form micelles for absorption. Fat malabsorption has many clinical consequences including failure to thrive in infants and children. In both children and adults fat malabsorption results in steatorrhea, fat soluble vitamin and essential fatty acid deficiencies and a risk of neurological deficits related to such nutrient deficiencies. 

Hereditary causes of pancreatic lipase insufficiency include cystic fibrosis, Shwachman syndrome, Johanson-Blizzard syndrome, Pearson pancreatic and bone marrow syndrome and isolated lipase deficiency. Cystic fibrosis is by far the most common inherited deficiency and Shwachman syndrome the next more common. Acquired causes of lipase insufficiency may result from chronic enteropathy such as celiac disease, and this usually resolves once the celiac disease is treated. Surgical excision of pancreas tissue is rare in children, and more common in adults with pancreatic cancer and other conditions. Chronic pancreatitis is relatively rare in children and common in adults. The chronic inflammation results in destruction of the pancreatic tissue, replacement with fibrotic tissue and loss of enzyme secretion. 

Patients with cystic fibrosis (CF) and pancreatic insufficiency (PI) often experience chronic malnutrition associated with dietary fat malabsorption, gastrointestinal inflammation, intestinal dysmotility and mucosal perturbations. Harris et al. [1] were the first to establish the relationship between PI and malnutrition in children with CF over 40 years ago. Growth and nutritional status in children and adults with CF are related to lung disease progression, PI and to nutrient intake and absorption [2, 3, 4]. Although the median predicted age of survival for patients with CF has dramatically increased from 14 years in 1969 to 27 years in 1986 to 38 years in 2010 [5], nutrition-related growth faltering in children remains at an unacceptably high rate. 

Assessing the degree of fat malabsorption in patients with CF and PI will support individualized enzyme therapy and nutritional intervention. The coefficient of fat absorption (CFA) obtained from the 72-hour stool and dietary intake collection method is accepted as a reliable fat malabsorption assessment [6, 7, 8]. The variable accuracy of this test is dependent on a 3-day complete stool collection together with consumption of a moderate to high fat diet with precise documentation. The coefficient of fat absorption is calculated from weighed food records and complete 72-hour stool as the average daily dietary fat intake (g) minus the average daily fecal fat excretion (g) divided by the average daily dietary fat intake (g). Despite the analytical accuracy of the gravimetric method to assess the total fat content of the stool [9], this test is prone to errors due to inaccurate dietary intake, incomplete stool collections and day-to-day variation in dietary fat and fecal excretion [10]. In addition, the aesthetic issues of stool collection, storage, shipping, homogenization and analysis make this test undesirable for patients, families and laboratory workers. This diagnostic and clinical test is poorly accepted and rarely used by patients, care providers, and investigators [11, 12]. The fecal elastase 1 test from a spot stool collection (either monoclonal or polyclonal test) is utilized in clinical and research settings to determine pancreatic status, but is useful only to determine pancreatic insufficiency vs. sufficiency and not the degree of fat malabsorption which varies across individuals with CF and other conditions with malabsorption [13]. 

We have developed a Malabsorption Blood Test (MBT) as a potentially more accurate, specific, and an acceptable alternative to the 72-hour stool and diet derived coefficient of fat absorption in patients and research subjects with CF and PI and with other fat malabsorption diagnoses. The formulation probe of the MBT consists of simultaneous oral doses of two odd-chained fatty acids, pentadecanoic acid (PA), a free fatty acid, and triheptadecanoic acid (THA), a triglyceride composed of glycerol with three heptadecanoic (HA) fatty acids are given. PA (C15:0) and HA (C17:0) are both odd-length saturated fatty acids that are present in small amounts in dairy products and synthesized by humans. PA, the free fatty acid, is absorbed without hydrolysis by pancreatic lipase, while THA, a triglyceride, requires hydrolysis before HA is absorbed. The extent of HA absorption following administration of the MBT reflects the degree of fat malabsorption due to deficits in pancreatic lipase activity, while PA absorption is unaffected. Therefore, PA and HA pharmacokinetic characteristics in combination, and particularly the HA/PA ratio, can be used to evaluate pancreatic-based fat malabsorption in patients and research subjects. These two odd-chained fatty acids were selected for the MBT for the following reasons: 1) they are found in small amounts only in dairy foods; 2) the vast majority of individuals have very low serum concentrations of PA and HA; 3) they are safe to administer in larger doses than typically found in the diet; and 4) they are easily identified in the serum by well-established laboratory methods, and absorption pharmacokinetics can be readily described. 

We have conducted a pilot investigation to examine the potential of the MBT to (1) assess malabsorption in subjects with CF and PI compared to healthy volunteer controls and (2) judge the effectiveness of pancreatic lipase inhibition as proof of mechanism for the combined administration of PA and HA to discriminate pancreatic enzyme-based fat malabsorption. The results of these initial experiments are expected to guide the MBT development as a diagnostic for malabsorption in subjects with CF and other pancreatic based malabsorption disorders. 

## Methods 

### Clinical studies 

Two MBT protocols were conducted in healthy subjects and subjects with CF and PI to characterize the absorption kinetics of PA and HA administered orally. The aim of the Lipase Inhibitor Study was to demonstrate that the MBT can detect fat malabsorption in healthy adult subjects when the MBT was administered with the pancreatic lipase inhibitor medication Orlistat (Xenical^®^, Roche Pharmaceuticals, Nutley, NJ, USA). Orlisat is a reversible inhibitor of pancreatic lipase, and is FDA approved for treatment of obesity in adults. The baseline MBT doses were 2.5 g of PA and 8.0 g of THA. Wolk and colleagues [14] have estimated as average daily intake of PA and HA of 0.22 and 0.15 g, respectively, in adults who consume dairy products. The experimental doses of PA and THA chosen for this study represent exposure to these fats 10-fold higher than normal dietary intake, providing a strong systemic signal. Subjects were then given a 250 mg capsule of Orlistat with each meal and snack for 5 days, and a follow-up MBT was conducted on Day 5. Subjects 18 years of age and older and of both sexes were eligible. Subjects were excluded if they had any chronic illness known to affect nutrient absorption, a body mass index (BMI) < 21 or > 30 kg/m^2^, diabetes mellitus, or disorders associated with altered energy metabolism or gastrointestinal motility. Subjects who were taking blood lipid lowering drugs were not eligible. 

The aim of the CF Pharmacokinetics Study was to determine PA and HA pharmacokinetics in subjects with CF and PI with and without their prescribed pancreatic enzyme medication. The baseline MBT doses were 2.5 g of PA and either 5.0 g or 8.0 g of HA on two separate occasions. In random order, the MBT was administered with or without the subject’s normal evening meal dose of pancreatic enzymes. The two MBT tests were performed at least 7 days apart. Patients with CF and PI who were 8 years of age or older and in an otherwise state of good health were enrolled. The diagnosis of CF was based upon clinical symptoms and duplicate quantitative pilocarpine iontophoresis sweat tests with Na and Cl values > 60 mEq/l. PI was determined based on clinical symptoms and laboratory values including 72-hour fecal fat analysis with < 93% absorption or a stool trypsin value of < 80 µl/g. Subjects were ineligible if they have an FEV_1%_ predicted of < 40%, a history of fibrosing colonopathy or bowel resection > 10 cm. Subjects with diabetes mellitus or disorders associated with altered energy metabolism, or illnesses that affect the gastrointestinal tract, were not eligible for inclusion. 

For both the Lipase Inhibitor and CF Pharmacokinetics Study, subjects were instructed to eat their typical diet for 2 days prior to MBT dosing. 24 hours prior to MBT administration, subjects refrained from consuming alcohol, dairy products and non-routine physical activity. A 12-hour fast was standard prior to MBT. Informed consent was obtained from adult subjects (18 years old or older) and guardians of children and assent from children ages 8 – 17 years. The study protocols were approved by The Children’s Hospital of Philadelphia Institutional Review Board. 

### Malabsorption blood test 

The MBT test meal was mixed in a uniform fashion, with the two experimental fats (2.5 g of PA and either 5 g or 8 g of THA) blended into a test meal composed of 64 g chocolate Scandishake (Axcan Scandipharm, Birmingham, AL, USA), 6 oz soy milk, and 10 ml microlipids (NestleHealth Science, Vevey, Switzerland). Approximately 8 oz of the MBT test meal containing 550 calories, 32 g fat, and 52% of calories from fat was administered to study subjects. The test meal was designed to be similar to a dinner meal in caloric and fat content for a patient with CF and PI. 

Subjects arrived in early morning after the 12 hour fast, and height and weight measurements were obtained using standard research methods [15]. Prior to administration of the MBT test meal, blood sample was collected at baseline and an indwelling catheter was placed for subsequent blood draws at 1, 2, 3, 4, 5, 6, 7 and 8 hours post ingestion of the MBT test meal for quantification of serum PA and HA. Immediately after the initial blood draw, subjects consumed the MBT test meal within 5 minutes. Given the high fat content of the MBT test meal, subjects with CF “on-enzymes protocol” took their prescribed evening meal pancreatic enzyme dose. For subjects with CF “off-enzymes protocol”, subjects refrained from taking pancreatic enzymes at the evening meal of the day prior to the test, and the MBT test meal was consumed without pancreatic enzymes. During the eight-hour protocol period, subjects were offered non-caloric and non-caffeinated beverages. At Hour 5, subjects were offered a ad libitum 1,000 kcal, low fat (12 g) lunch meal. Subjects with CF “on-enzymes protocol” took their prescribed enzyme dose with lunch, while those “off-enzymes protocol” took no enzymes. 

Plasma samples were analyzed using a standardized gas chromatographic (GC) method. Total lipids were extracted from 200 µl of plasma following the method of Bligh and Dyer [16]. At the time of extraction tritridecanoate was added as an internal standard. Following methylation of the fatty acids in the total lipid extract [17], fatty acid methyl esters were extracted into isooctane and 1 µl was injected into the GC column for analysis of the fatty acids and quantification of PA and HA. The fatty acid composition was determined by GC analysis using a Hewlett Packard 5890 series II gas chromatograph with a programmable cool on-column capillary inlet, flame ionization detector (FID), and HP7673 auto sampler/injector. Chromatographic data collection and analysis was via a serial connection to a computer running ChromPerfect Spirit™ chromatography data system (Justice Laboratory Software, Denville, NJ). The column used was a CP-Sil 88 for FAME, 100 m (L) × 0.25 mm (ID) × 0.25 µm (film thickness) (Agilent Technologies, Inc., Santa Clara, CA, USA) with a 1 m (L) × 0.53 mm (ID) deactivated precolumn. The carrier gas was hydrogen at a constant head pressure of 20 psi with helium as make-up gas. Oven temperature was programmed to begin at 90 °C for 0.5 minutes, increase 10 °C per minute to 150 °C; increase at 2.5 °C to 200 °C; increase at 1.5 °C to 220 °C then hold for 20 minutes. The FID was maintained at 230 °C. There was a 5-min equilibration period between runs. Total analysis time was 65 minutes per sample. Calibration curves were based on injections of methyl tridecanoate, methyl PA, and methyl HA injections. Inter-assay variability (%CV) for the measurement of PA in samples with low, medium and high concentrations (1.30, 2.99, and 6.70 mg/dl) was 2.9%, 2.6%, and 3.1%, respectively. Inter-assay variability for the measurement of HA (0.56, 1.29, 3.05 mg/dl) in the same samples was 2.6%, 4.0%, and 3.9%, respectively. 

### Pharmacokinetic and statistical analysis 

A moment-based pharmacokinetic analysis was performed based on non-compartmental methodology for each study using WinNonLin version 9.1 (Pharsight, Cary, NC, USA). Baseline (C_0_) and maximum (C_max_) plasma concentrations were calculated. Area under the curve from Time zero to 8 hours (AUC) was calculated using the linear trapezoid method. All plasma concentrations were normalized to the baseline concentration prior to analysis. Descriptive statistics (mean, standard deviation, 95% CI) were calculated for each treatment group for both studies. Pharmacokinetic parameters were compared between treatment groups using a paired t-test or Wilcoxon signed-rank test as appropriate. To describe HA exposure relative to that of PA, the ratio of the HA to PA C_max_ and AUC was calculated for each subject after molar transformation and dose-normalization of exposure metrics. The difference of means and 95% confidence intervals (CI) between treatment groups were also calculated. The 95% CI for AUC ratios were calculated via bootstrapping. 

## Results 

Subject characteristics for both the healthy subjects in the Lipase Inhibitor Study and the subjects with CF in the CF Pharmacokinetic Study are presented in Table 1, both for the total samples and separately by gender. 60% of the healthy subjects were female and 50% of the subjects with CF. Subjects with CF were younger and had lower body weight and BMI. There were no serious adverse events either observed or reported. 

### Lipase inhibitor study in healthy subjects 

**Figure 1:**
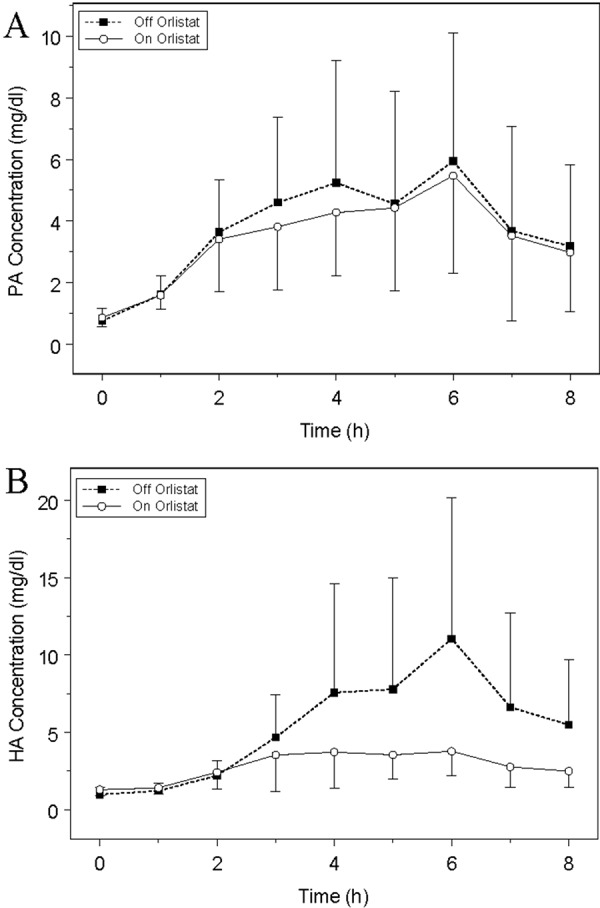
Mean (A) PA and (B) HA concentration-time profiles resulting from 15 healthy subjects administered 2.5 g of PA and 8 g of THA in a MBT dosed with and without Orlistat.

Mean concentration-time profiles resulting from the 15 healthy subjects enrolled in the Lipase Inhibitor study are shown in Figure 1. Profiles for both fats were characterized by variability and extended absorption periods relative to the 8-hour sampling window. PA and HA plasma concentrations did not return to baseline values within the eight hours observation. PA concentrations were comparable in healthy subjects untreated and after Orlistat treatment. HA concentrations showed 65 – 71% reduction with Orlistat treatment depending upon the measure of fat absorption. 

Results of the noncompartmental pharmacokinetic analysis are shown in Table 2. PA pharmacokinetics were comparable untreated subjects and after Orlistat treatment. Mean baseline concentrations (C_0_) were not significantly different between untreated and treated protocols (1.02 and 0.763 mg/dl, respectively). Similarly, C_max_ and AUC for PA did not differ between the untreated and treated protocols. 

Following Orlistat treatment, HA absorption was significantly reduced. The mean C_max_ was 11.19 (6.38, 16.0) mg/dl for untreated and decreased to 3.28 (1.93, 4.64) mg/dl after Orlistat treatment (p = 0.0004). Similarly, AUC was 36.5 (19.7, 53.2) mg/dl·h for untreated, and 12.8 (8.03, 17.5) mg/dl·h after Orlistat treatment (p = 0.001). There was a 71% reduction in C_max_ and a 65% reduction for AUC in HA concentration after Orlistat treatment. 

**Figure 2:**
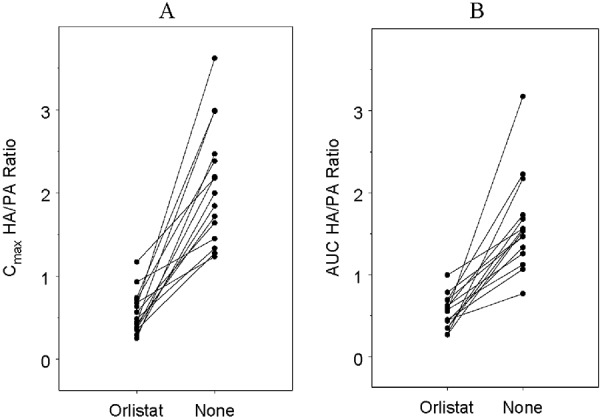
C_max_ (A) and AUC (B) HA/PA ratios by treatment for 15 healthy subjects enroled in the Orlistat study.

Individual C_max_ and AUC HA/PA ratios are shown in Figure 2. For C_max_, the mean HA/PA ratio decreased from 1.62 (1.01, 2.24) to 0.56 (0.31, 0.81) with Orlistat treatment, while AUC decreased from 1.30 (0.82, 1.78) to 0.53 (0.360, 0.70). This marked decrease in the absorption of HA relative to PA indicates the fat malabsorption specific to lipase inhibition. 

### CF pharmacokinetics study 

**Figure 3:**
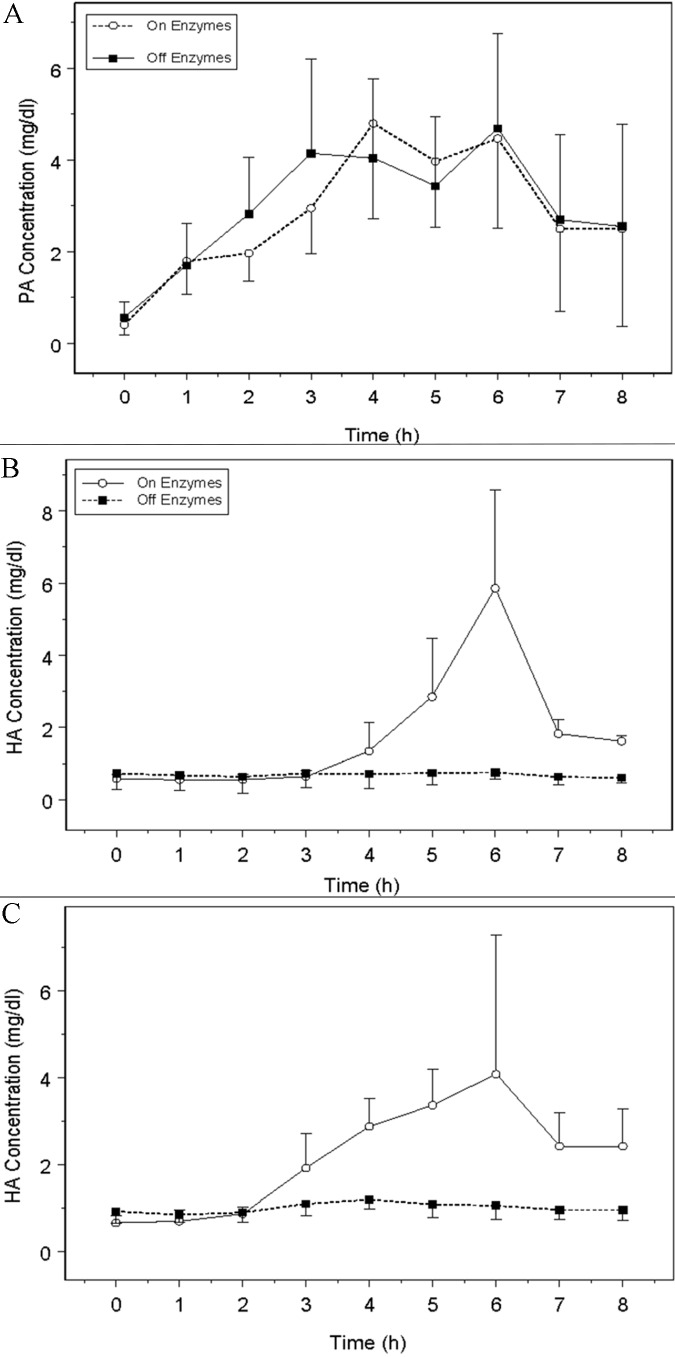
PA and HA concentration-time profiles resulting from 6 subjects with CF administered a MBT dosed with and without pancreatic enzymes. A: PA concentrations for 6 subjects dosed 2.5 g PA. B: HA concentrations for 3 subjects dosed 5 g of THA. C: HA concentrations for 3 subjects dosed 8 g of HA.

Mean concentration-time profiles for the six subjects enrolled in the CF study are shown in Figure 3. PA concentration-time profiles were similar to those observed in the healthy subjects. PA profiles were comparable in the subjects with CF for both the “on enzyme” and “off enzyme” protocols. HA concentrations after MBT administration in subjects with CF on the “off enzyme” protocol did not increase from baseline values, but they were significantly increased during the “on enzyme” protocol. Even with the increase in HA “on enzymes”, the levels for subjects with CF were lower than those observed in healthy subjects. 

Results of the noncompartmental pharmacokinetic analysis for the CF study are presented in Table 3. PA pharmacokinetics were similar for subjects with CF on both the “on enzyme” and “off enzyme” protocols (C_max_, p = 0.69; AUC, p = 0.84). HA absorption was minimal in subjects with CF when the MBT was administered in the “off enzyme” protocol, as reflected in the low values of C_max_ and AUC. In contrast, for the “on enzyme” protocol, HA exposure was increased after administration of the MBT. The average AUC ratios were 0.012 and 0.032 for the 5 mg and 8 mg THA doses respectively for the “off enzyme treatment”. The ratios increased to 0.53 and 0.71 for the 5 mg and 8 mg dose when the MBT was administered with pancreatic enzymes. 

**Figure 4:**
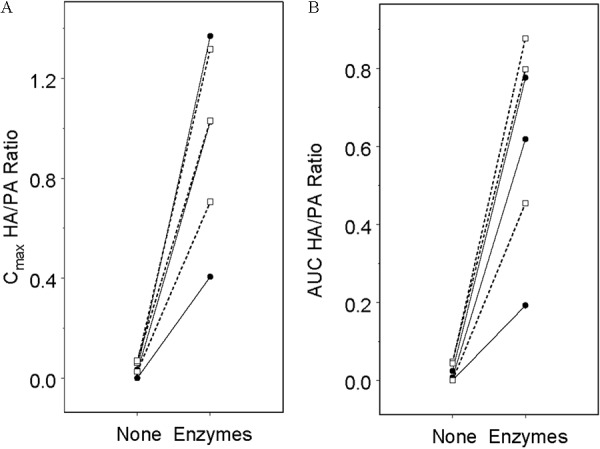
C_max_ (A) and AUC (B) HA/PA ratios by treatment for 6 subjects with CF enroled in the CF Enzyme study. Solid lines with closed circles represent 3 subjects administered 5 g of THA. Dashed lines with open squares represent 3 subjects administered 8 g of THA.

Individual C_max_ and AUC HA/PA ratios are shown in Figure 4. For both measures of exposure, enzyme administration resulted in a significant increase in HA concentrations relative to PA. C_max_ HA/PA ratios increased from 0.020 (–0.243, 0.283) to 0.921 (0.199, 1.640) in subjects administered 5.0 g THA and increased from 0.047 (–0.247, 0.341) to 0.731 (0.130, 1.330) for subjects administered 8.0 g THA. Similarly, AUC HA/PA ratios in subjects dosed 5.0 g THA, increased from 0.0076 (–0.093, 0.108) to 0.495 (0.218, 0.772), while in subjects dosed 8.0 g THA increased from 0.034 (–0.087, 0.154) to 0.539 (0.242, 0.835) with supplemental enzyme dosing. 

## Discussion 

The assessment of malabsorption is a critical concern for people with lipase-based pancreatic insufficiency resulting from inherited disorders such as CF and also from acquired insufficiency due to sequelae from chronic diseases such as celiac disease or pancreatitis. Assessing the pattern of fat malabsorption in people with PI is needed for clinical research and clinical care. These pilot and feasibility proof-of-concept studies demonstrate potential clinical and research value of the MBT in patients with CF and PI and those with other lipase-based fat malabsorption conditions. 

The existing standard of care for assessment of fat malabsorption is problematic. Several alternative tests have been explored but all have significant limitations and have failed to gain wide acceptance as accurate or practical alternatives to the 72-hour stool and diet collection. The acid steatocrit test utilizes a small (~ 5 g) stool specimen which is centrifuged to separate the acidified fat from the solid stool [18, 19]. Correlation with the 72-hour stool and diet collection only occurs when homogenized-pooled stool is used. Therefore requirement for a pooled stool collection offers no added benefit to the patient and family, and the results were not well correlated with the 72-hour collection. Triolein breath tests [20, 21] are non-invasive tests utilizing small amounts of dietary fat labeled with stable or radioactive isotopes. Fat malabsorption is predicted by the labeled CO_2_, exhaled during respiratory gas exchange and is an indicator of the absorbed dietary fat. The data may be used to qualitatively monitor response to pancreatic enzyme therapy in known pancreatic insufficiency [22, 23]. When compared with 72-hour stool and diet collection, breath tests had up to 80% sensitivity but only 45% specificity. Since this is a breath test, there is also the assumption that the subject’s pulmonary status is normal or near normal, and may limit its use in people with CF, most of whom have a continuous slow decline in pulmonary function and occasional acute pulmonary exacerbations. ^14^C is a radioactive compound and rarely used in children. ^13^C, the stable isotope, is safe for use in children as well as in adults, however costs of the substrate and mass spectrometry instruments and skills and time required for analysis are high. The placement and maintenance of an in-dwelling catheter for intravenous sampling will probably need to be reserved to centers with expertise in child health care. 

Due to these limitations, isotope breath tests have failed to become widely accepted or utilized in CF research or clinical care. 

We propose herein an oral-based fat malabsorption test which uses plasma profiling of odd-chained fatty acids (THA and PA) to discriminate malabsorption behavior. Our initial proof-of-concept studies have been conducted to evaluate its clinical potential in the categorization of children with CF. Improved treatment of PI and related malnutrition would benefit from the development of more reliable, specific, and quantitative tests for the evaluation of malabsorption. Novel pancreatic enzyme products or modifications of current products require evaluation by a more stringent research quality test for malabsorption. Additionally, the current “one size fits all” therapy for administering pancreatic enzyme replacement therapy may not be ideal for achieving maximum effectiveness with enzyme medication. Furthermore, very high dose enzyme medication in CF poses a safety risk for the development of fibrosing colonopathy [24, 25]. Assessing the degree of fat malabsorption will ultimately provide informative guidance for both enzyme therapy and nutritional intervention in children with CF and PI and likely patients with other diseases. 

The results from the Lipase Inhibitor and CF Pharmacokinetics Studies demonstrate the ability of the MBT to recognize fat malabsorption in subjects with PI. Since baseline serum concentrations are minimal [14] and endogenous synthesis is absent in humans, PA and HA are ideal markers for use in the MBT. The MBT demonstrates a significant reduction in absorption of HA with pancreatic lipase inhibition (Orlistat use in healthy subjects) and in the setting of a reduction in pancreatic lipase activity (CF study with no enzyme dose). As expected, both the amount of HA absorbed, based upon AUC and C_max_ values and the ratio of HA absorption to PA absorption were significantly reduced. As predicted, PA absorption, the free fatty acid, was not affected by lack of pancreatic lipase in CF nor the pharmacologically induced PI with Orlistat dose. This lack of response for PA supports our hypothesis for no change in absorption for this free fatty acid and, therefore, PA serves as an important quality control for the MBT performance. 

In these pilot studies, the individual lipid profiles demonstrated inter-subject as well as intra-subject variability in PA exposure. This result was not unexpected given the nature of the absorption process, and CF-related alterations of the gastrointestinal tract [11]. These characteristics of fat absorption support the use of PA as a control to which HA absorption is adjusted. Additional studies are required to demonstrate the clinical and research utility of the MBT. Pancreatic enzyme titration studies will evaluate the ability of the MBT to characterize changes in the patterns of malabsorption in response to altering enzyme doses. Direct comparison of the MBT to the 72-hour diet and fecal fat method will be informative. Future studies are needed to evaluate the MBT applicability in other disease settings such as chronic pancreatitis where malabsorption is observed. There is also a potential for using the MBT to evaluate the biopharmaceutic characteristics of poorly absorbed, oral pharmaceuticals. 

The overall development aim of the MBT is to create an acceptable, accurate, reliable and specific alternative to the 72-hour stool and diet collection to determine the changes in fat absorption with alterations in disease severity (i.e., transition from mild to severe fat malabsorption) and to evaluate enzyme dose optimization in individual patients and in research subjects. 

The MBT may have utility in clinical trials demonstrating the impact of manufacturing procedure changes in existing enzyme products and in demonstrating efficacy in new products under review for approval. A series of studies are needed to further develop and demonstrate the clinical utility of the MBT. A direct comparison with the “gold standard” 72-hour stool and diet collection in detection of the degree of fat malabsorption with PI is indicated. Other studies needed include MBT reproducibility studies in subjects with CF and studies to determine the stool loss of the PA and THA fatty acids. Pancreatic enzyme titration studies would be necessary to assess the ability of the MBT to characterize changes in the degree of fat malabsorption in patients with PI in response to altering enzyme doses and also in response to the timing of enzyme administration (before, during and after meals). Differences between subjects with CF and healthy controls in gastric emptying and small bowel transit may exist and should be explored as this may affect fat absorption and have an impact on the pharmacokinetic patterns of the MBT. 

## Conclusions 

This pilot study indicates that the MBT holds promise to provide a more accurate, specific and acceptable alternative method to the 72-hour stool collection for quantifying the pattern of dietary fat malabsorption. The ability of the MBT to respond to the changes in fat absorption in healthy subjects using a lipase inhibitor and in subjects with CF while on or off enzyme therapy, indicates that MBT detects pancreatic-based fat malabsorption in a variety of contexts. Future studies are needed to evaluate the MBT across age groups and other pancreatic insufficiency-related diagnoses, and also in comparison to other methods designed to detect fat malabsorption. 

## Acknowledgments 

This study was supported by the Cystic Fibrosis Foundation, Bethesda, MD, and the Clinical Translational Research Center (CTRC), Foederer Foundation and Nutrition Center at Children’s Hospital of Philadelphia (CHOP), Philadelphia, PA. The CTRC is supported by Grant Number UL1RR024134 from the National Center for Research Resource. The content is solely the responsibility of the authors and does not necessarily represent the official views of the National Center for Research Resources or the National Institutes of Health. We also gratefully acknowledge the subjects with CF and their families who participated in this study, and the healthy volunteers. Finally, we also acknowledge the assistance of Kate Temme, MD who participated in data collection for this study while at CHOP, and Michael Phillips, PhD who, with his knowledge and expertise in lipid metabolism, provided us with consultation and assistance in both protocol development and interpretation of results. 

## Conflicts of interest 

There is no actual competing interest related to this work. No authors have any financial or personal relationships with other people or organizations that could have inappropriately influence this research. Some of these data were used after this study was completed for US Patent #7,402,405 BZ. 

**Table 1:** Demographic summary for the lipase inhibitor and CF pharmacokinetic studies.

		Lipase Inhibitor Study (n = 15)			CF Pharmacokinetic Study (n = 6)		
		Age years	Weight kg	BMI kg/m^2^	Age years	Weight kg	BMI kg/m^2^
Total	Mean	30.4	77.9	26.1	15.0	48.6	20.1
	SD	8.5	10.8	3.2	2.5	5.5	2.4
	Range	21 – 49	60.0 – 95.4	21.3 – 31.6	13 – 19	40.6 – 55.6	17.8 – 21.4
Male*	Mean	29.0	85.3	25.5	13.3	44.2	18.3
	SD	10.4	9.3	3.0	0.6	3.5	0.9
	Range	21 – 49	71.2 – 95.4	21.3 – 28.4	13 – 14	40.6 – 47.6	17.8 – 19.3
Female	Mean	31.3	72.9	26.5	16.7	53.0	21.9
	SD	7.6	9.1	3.4	2.5	2.3	2.1
	Range	24 – 46	60 – 89.7	21.9 – 31.6	14 – 19	51.6 – 55.6	20.2 – 24.2

*Sample sizes: Lipase Inhibitor Study in Healthy subjects: n = 6 males, n = 9 females; CF Pharmacokinetic Study: n = 3 males, n = 3 females.

**Table 2:** Noncompartmental pharmacokinetic analysis results for 15 healthy subjects administered the MBT with and without Orlistat. tmax reported as the median and range.

Treatment	Fat		C_0_ (mg/dl)	t_max_ (h)	C_max_ (mg/dl)	AUC (mg×h×dl^–1^)
None	HA	Mean	0.979	5.5	11.2	36.4
		SD	0.429	[4 – 7]	8.68	30.2
		95% CI	(0.741, 1.22)		(6.38, 16.0)	(19.7, 53.2)
	PA	Mean	0.763	4	6.19	25.1
		SD	0.394	[2 – 6]	4.27	17.4
		95% CI	(0.545, 0.981)		(3.83, 8.55)	(15.5, 34.7)
Orlistat	HA	Mean	1.28	4.5	3.28	12.8
		SD	0.340	[3 – 6]	2.44	8.54
		95% CI	(1.07, 1.39)		(1.93, 4.64)	(8.03, 17.5)
	PA	Mean	1.02	4	5.26	21.6
		SD	0.674	[2 - 6]	3.14	11.8
		95% CI	(0.646, 1.39)		(3.52, 6.99)	(15.1, 28.1)

**Table 3:** Noncompartmental pharmacokinetic analysis results for 6 subjects with CF administered the MBT with 5 g of THA (n = 3) or 8 g THA (n = 3) with and without pancreatic enzymes. tmax reported as the median and range.

Treatment	Fat		C_0_ (mg/dl)	t_max_ (h)	C_max_ (mg/dl)	AUC (mg×h×dl^-1^)
None	HA: 5 g THA	Mean	0.733	5	0.123	0.177
		SD	0.462	[0 – 6]	0.157	0.232
		95% CI	(–0.414, 1.88)		(–0.267, 0.513)	(–0.401, 0.754)
	HA: 8 g THA	Mean	0.917	3	0.290	0.797
		SD	0.277	[3 – 6]	0.104	0.720
		95% CI	(0.227, 1.61)		(0.0306, 0.549)	(–0.991, 2.58)
	PA	Mean	0.673	4	5.54	21.3
		SD	0.327	[3 – 6]	1.96	5.98
		95% CI	(0.338, 1.01)		(3.53, 7.54)	(15.2, 27.4)
Enzyme	HA: 5 g THA	Mean	0.590	6	5.46	11.5
		SD	0.127	[6 – 6]	3.07	6.50
		95% CI	(0.274, 0.906)		(–2.16, 13.2)	(–4.65, 27.61)
	HA: 8 g THA	Mean	0.653	6	4.33	12.5
		SD	0.156	[5 – 8]	2.40	3.95
		95% CI	(0.265, 1.04)		(–1.65, 10.3)	(2.66, 22.3)
	PA	Mean	0.522	5	5.31	20.8
		SD	0.0763	[4 – 6]	2.36	6.45
		95% CI	(0.444, 0.600)		(2.90, 7.72)	(14.2, 27.4)
